# Transcriptome analysis in petals and leaves of chrysanthemums with different chlorophyll levels

**DOI:** 10.1186/s12870-017-1156-6

**Published:** 2017-11-15

**Authors:** Akemi Ohmiya, Katsutomo Sasaki, Kenji Nashima, Chihiro Oda-Yamamizo, Masumi Hirashima, Katsuhiko Sumitomo

**Affiliations:** 1 Institute of Vegetable and Floriculture Science, National Agriculture and Food Research Organization, Fujimoto 2-1, Tsukuba, Ibaraki 305-0852 Japan; 2 Institute of Fruit Tree and Tea Science, National Agriculture and Food Research Organization, Fujimoto 2-1, Tsukuba, Ibaraki 305-8605 Japan; 3 College of Bioresource Sciences, Nihon University, 1866 Kameino, Fujisawa, Kanagawa 252-0880 Japan

**Keywords:** Chlorophyll metabolism, Chrysanthemum (*Chrysanthemum morifolium* Ramat.), Gene expression, Petal color, Transcription factor

## Abstract

**Background:**

Chlorophylls (Chls) are magnesium-containing tetrapyrrole macromolecules responsible for the green color in plants. The Chl metabolic pathway has been intensively studied and nearly all the enzymes involved in the pathway have been identified and characterized. Synthesis and activity of these enzymes are tightly regulated in tissue- and developmental stage–specific manners. Leaves contain substantial amounts of Chls because Chls are indispensable for photosynthesis. In contrast, petals generally contain only trace amounts of Chls, which if present would mask the bright petal color. Limited information is available about the mechanisms that control such tissue-specific accumulation of Chls.

**Results:**

To identify the regulatory steps that control Chl accumulation, we compared gene expression in petals and leaves of chrysanthemum cultivars with different Chl levels. Microarray and quantitative real-time PCR analyses showed that the expression levels of Chl biosynthesis genes encoding glutamyl-tRNA reductase, Mg-protoporphyrin IX chelatase, Mg-protoporphyrin IX monomethylester cyclase, and protochlorophyllide oxidoreductase were well associated with Chl content: their expression levels were lower in white petals than in green petals, and were highest in leaves. Among Chl catabolic genes, expression of *STAY-GREEN*, encoding Mg-dechelatase, which is a key enzyme controlling Chl degradation, was considerably higher in white and green petals than in leaves. We searched for transcription factor genes whose expression was well related to Chl level in petals and leaves and found three such genes encoding MYB113, CONSTANS-like 16, and DREB and EAR motif protein.

**Conclusions:**

From our transcriptome analysis, we assume that a low rate of Chl biosynthesis and a high rate of Chl degradation lead to the absence of Chls in white chrysanthemum petals. We identified several candidate transcription factors that might affect Chl accumulation in chrysanthemum petals. Functional analysis of these transcription factors will provide a basis for future molecular studies of tissue-specific Chl accumulation.

**Electronic supplementary material:**

The online version of this article (10.1186/s12870-017-1156-6) contains supplementary material, which is available to authorized users.

## Background

Chlorophylls (Chls) are magnesium-containing tetrapyrrole macromolecules responsible for the green color in plants. Because Chls play a central role in light harvesting and energy transduction in photosynthesis, mature leaves contain a substantial amount of Chls [1]. Petals of many flowering plants contain Chls at early developmental stages [2]. As petals develop, Chl content decreases, and petals of fully opened flowers contain only trace amounts of Chls. The absence of Chls in petals is an important trait that enables flowers to be visually distinguished by pollinators against a background of leaves when the flowers are ready to be pollinated.

**Fig. 1:**
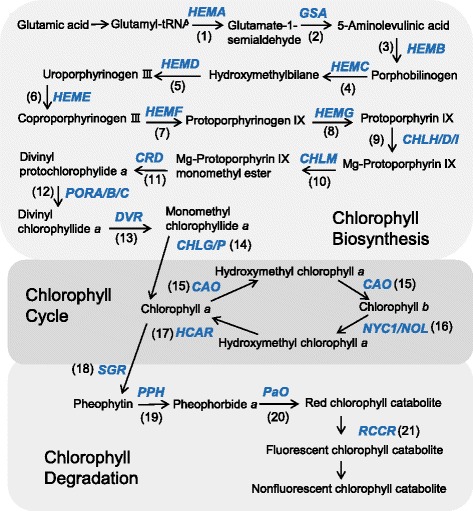
Schematic representation of Chl metabolic pathways in higher plants. Genes (italicized) encode the following enzymes: (1) glutamyl-tRNA reductase; (2) glutamate-1-semialdehyde 2,1-aminotransferase; (3) 5-aminolevulinate dehydrogenase; (4) porphobilinogen deaminase; (5) uroporphyrinogen III synthase; (6) uroporphyrinogen III decarboxylase; (7) coproporphyrinogen III oxidase; (8) protoporphyrinogen oxidase; (9) Mg-chelatase; (10) Mg-protoporphyrin IX methyltransferase; (11) Mg-protoporphyrin IX monomethylester cyclase; (12) protochlorophyllide oxidoreductase; (13) divinyl chlorophyllide *a* 8-vinyl-reductase; (14) Chl synthase; (15) chlorophyllide *a* oxygenase; (16) Chl *b* reductase; (17) hydroxymethyl Chl *a* reductase; (18) STAY-GREEN (Mg-dechelatase); (19) pheophytinase; (20) pheophorbide *a* oxygenase; (21) red Chl catabolite reductase

The Chl metabolic pathway can be divided into three distinct phases (Fig. 1) [3–5]: (1) biosynthesis of Chl *a* from glutamate; (2) interconversion between Chl *a* and *b* (Chl cycle); and (3) degradation of Chl *a* into a non-fluorescent Chl catabolite. Nearly all enzymes involved in the pathway are identified and characterized. In higher plants, synthesis and activity of these enzymes are tightly regulated in tissue- and developmental stage–specific manners.

There is increasing evidence that the Chl biosynthesis pathway is transcriptionally regulated in a tissue-specific manner [4, 6]. Several transcription factors have been identified as negative or positive regulators of the Chl biosynthesis pathway. GOLDEN2-LIKE (GLK), LONG HYPOCOTYL5 (HY5), and GATA, NITRATE-INDUCIBLE, CARBON-METABOLISM INVOLVED (GNC) regulate Chl biosynthesis in leaves [7–9], whereas GNC-LIKE (GNL)/CYTOKININ RESPONSIVE GATA FACTOR1 (CGA1) enhances Chl biosynthesis in petals and stamens [9]. PHYTOCHROME-INTERACTING FACTOR 1 (PIF1) and PIF3 inhibit the biosynthesis of protochlorophyllide by interacting with photoreceptors in leaves in the dark [10]. Chl degradation is also regulated at the transcriptional level. Recently, ORE1 and ANAC046 have been shown to increase the expression of Chl catabolic genes [11, 12]. Changes in the expression of genes for these transcription factors influence Chl content in Arabidopsis leaves, but limited information is available about the regulation of Chl metabolism in non-photosynthetic tissues.

As mentioned above, it is a distinct disadvantage for insect-pollinated flowers to accumulate Chls in petals. Therefore, even if a mutation that results in green flowers occurs, such plants may be eliminated from their natural habitat. In contrast, in ornamental plant breeding, green-flowered mutants are preferably selected and developed as cultivars. In this study, we used white- and green-flowered cultivars of chrysanthemum (*Chrysanthemum morifolium* Ramat.) to investigate the mechanisms that regulate Chl content in petals. We compared the expression profiles of genes related to Chl metabolism between these cultivars and searched for genes whose expression levels are positively or negatively associated with Chl content. We also searched for genes encoding transcription factors whose expression is regulated in coordination with Chl content in petals. From these data, we identified candidate transcription factors controlling Chl accumulation in chrysanthemum petals.

## Methods

### Plant materials

The chrysanthemum (*Chrysanthemum morifolium* Ramat.) cultivars Feeling White (FW), Feeling Green (FG), and Feeling Green Dark (FGD) (Japan Agribio Co., Shizuoka, Japan) were grown under natural daylight in a greenhouse at our institute (Tsukuba, Ibaraki, Japan). FGD and FW were bud sports arising from FG. Other white- and green-flowered chrysanthemum cultivars were purchased from the local market in Tsukuba. Petals of ray florets and the 3rd visible leaves from the top were harvested, immediately frozen in liquid nitrogen, and stored at −80 °C until use.

### Microarray analysis

We previously constructed a chrysanthemum expressed sequence tag (EST) database with 213,204 contigs [13]. Short contigs (<100 bp) and no-hit contigs (<197 bp) were omitted, and 176,026 contigs were chosen for custom oligonucleotide array design. Oligonucleotides of about 60 bases representing each EST were designed by Takara Bio (Ohtsu, Shiga, Japan) as described by Ohba et al. [14]. Oligonucleotides were synthesized on a glass surface (4 × 180 k) with SurePrint technology (Agilent Technologies, Palo Alto, CA, USA).

For microarray analysis, we chose petals at S2 because the difference in chlorophyll content among FW, FG, and FGD became evident at this stage. Total RNA was extracted from ray floret petals of FW, FG, and FGD at S2, and mature leaves of FG using Trizol reagent (Thermo Fisher Scientific, Waltham, MA, USA) and an RNeasy Mini Kit (Qiagen, Hilden, Germany). RNA integrity was evaluated using an Agilent 2100 Bioanalyzer (Agilent Technologies). Total RNA samples (100 ng) were used as starting materials. Three biological replicates were examined by the one-color method according to Nashima et al. [15]. Spot signal values were calculated with Agilent Feature Extraction version 9.1 software. Hierarchical clustering using Pearson correlation was performed with the tree-clustering tool of the Subio platform (Subio Inc., Kagoshima, Japan). The datasets for hierarchical clustering were normalized using a low signal cutoff (mean raw signal <100), log_2_-transformation, global normalization, and centering.

Gene ontology (GO) categorization was performed according to the to the annotation of the Arabidopsis gene corresponding to each probe [13]. GO terms were obtained from The Arabidopsis Information Resource (TAIR, http://www.arabidopsis.org/) (ATH GO GOSLIM, updated 2013 May 15).

### Quantitative real-time PCR analysis

Quantitative real-time PCR (RT-qPCR) was performed as described previously [16]. cDNA for each gene was amplified by RT-PCR, cloned into the pGEM-T Easy Vector (Promega, Madison, WI, USA), and sequenced. Primers for RT-qPCR were designed from the sequences (Additional file 1 Table S1). Each plasmid was serially diluted 10-fold and used for a standard curve assay. The transcript copy number was determined by relating the RT-qPCR signal for each gene to a standard curve. The mRNA levels were calculated relative to that of *actin* (IABW01167629), which was constitutively expressed in leaves and petals as indicated by our microarray data. Analysis was performed in biological triplicate, and statistical significance was analyzed by Tukey–Kramer multiple-comparison test (*P* < 0.05).

### Chlorophyll analysis

Tissues were ground into powder in liquid nitrogen and extracted with acetone. The samples were centrifuged at 10,000×*g* for 10 min, and the supernatants (80 μl) were mixed with 20 μl of water. Pigments were analyzed by high-performance liquid chromatography (HPLC; X-LC, Jasco, Tokyo, Japan) using a reversed-phase column (Symmetry C8, 150 × 4.6 mm; Waters, Milford, MA, USA) according to Zapata et al. [17]. The analysis was performed in biological triplicate.

### Transmission electron microscopy

Tissues were cut into small pieces (approximately 1 mm^3^), fixed, dehydrated, and embedded in Quetol 651 (Nisshin EM Co., Tokyo, Japan) as described previously [12]. Ultrathin sections were cut with a diamond knife on an ultramicrotome (Ultracut UCT, Leica Microsystems, Wetzlar, Germany). Sections were picked up on copper grids, stained with uranyl acetate and lead citrate, and observed under a transmission electron microscope (TEM) (JEM-1200EX; JEOL Ltd., Tokyo, Japan) at an acceleration voltage of 80 kV. Digital images were taken with a CCD camera (Veleta; Olympus Soft Imaging Solutions GmbH, Münster, Germany).

## Results

### Chlorophyll content in petals and leaves

**Fig. 2:**
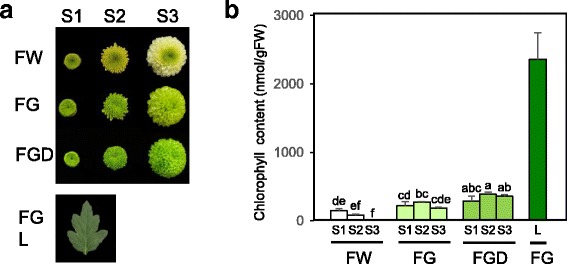
Chl content in petals and leaves of chrysanthemums. **a** Photographs of flowers of FW (Feeling White), FG (Feeling Green), and FGD (Feeling Green Dark) and an FG leaf (L). Flower development was divided into three stages: S1, 2–3 days after calyx opening; S2, ray florets at the outermost part of the capitulum begin to open; and S3, approximately 90% of ray florets are open. **b** Chl content in petals at different developmental stages and in mature leaves. Mean values (± SD) of three biological replicates are shown. The differences among petals (FW, FG, and FGD) were analyzed by Tukey–Kramer multiple-comparison test. Different letters indicate significant differences at *P* < 0.05

To identify genes coordinately expressed with Chl content, we used ray floret petals and leaves containing different Chl levels. In the white-flowered cultivar FW, small amounts of Chls accumulated in petals at the early developmental stage (S1) (Fig. 2). At the late stage (S3), Chl content decreased to extremely low levels. Larger quantities of Chls were detected in petals of the green-flowered cultivars FG and FGD than in those of FW especially at S3 (Additional file 4 Figure S2). At S3, FGD petals contained more Chls than FG and FW petals. At this stage, Chl content in FG leaves was 12.85 times that in FG petals and 6.56 times that in FGD petals. Because there was no significant difference in leaf Chl content among FW, FG, and FGD (Additional file 2 Figure S1), leaves of FG were used for further study.

### Gene ontology classification of differentially expressed genes

**Fig. 3:**
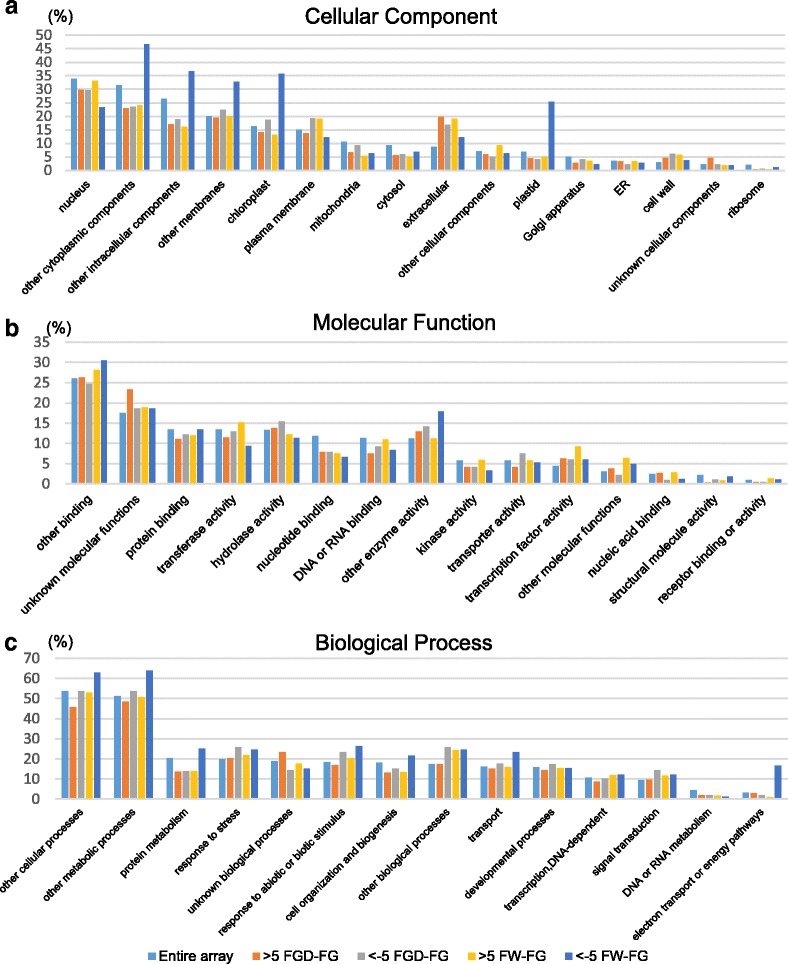
Gene ontology classification of genes differentially expressed in FW, FG, and FGD petals. Expression of genes in stage 2 petals was compared between FW and FG and between FGD and FG. Genes were classified into categories (cellular component, molecular function, and biological process) after being mapped to the Gene Ontology database. The *y*-axis indicates the percentage of genes in each functional category, calculated from the number of genes whose expression in FW or FGD petals was >5 times greater or lower than that in FG petals

Using microarray data, we extracted genes with the expression levels in FW or FGD petals >5 times greater or lower than in FG petals and examined the distribution of gene ontology terms (Fig. 3). In the cellular component category, genes associated with “plastid”, “chloroplast”, “other cytoplasmic component”, “other intracellular component”, and “other membrane” were highly enriched among the downregulated genes in FW petals. In the biological process category, genes associated with “electron transport or energy pathways”, which include photosynthesis-related genes, were also highly represented among the downregulated genes in FW petals. No marked differences were found in the molecular function category.

### Microarray and RT-qPCR analyses of chlorophyll-related gene expression

The chrysanthemum custom oligonucleotide array constructed in this study covered most genes involved in Chl metabolism (Additional file 3 Table S2). Microarray analysis was performed using this array and the expression profiles of selected genes of interest were further analyzed using RT-qPCR for a wider range of developmental stages of each tissue.

#### Chl biosynthesis

**Fig. 4:**
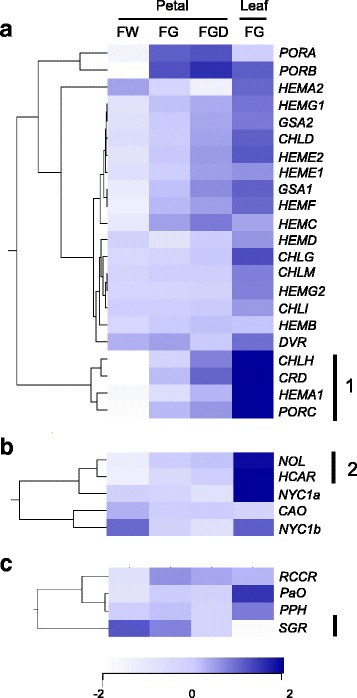
Overview of expression profiles of genes related to (**a**) Chl biosynthesis, (**b**) Chl cycle, and (**c**) Chl degradation in petals and leaves of chrysanthemum. The microarray data was obtained from S2 petals of FW, FG, and FGD and from mature leaves of FG. The data was clustered with respect to the transcript profiles of genes, and dendrograms are shown on the left of the heat map. Data represent log_2_-normalized signal values of transcript levels, which are continuously mapped on the color scale provided at the bottom of the figure

Many of the genes involved in Chl biosynthesis showed higher expression in FG and FGD petals than in FW petals (Fig. 4a). In particular, genes in group 1, *CHLH* (encoding Mg-​protoporphyrin IX chelatase [Mg-chelatase] H subunit), *CRD* (encoding Mg-protoporphyrin IX monomethylester cyclase), *HEMA1* (encoding glutamyl-tRNA reductase), and *PORC* (encoding protochlorophyllide oxidoreductase C) showed significantly higher expression in FG and FGD petals than in FW petals; the highest expression of these genes was found in FG leaves (*P <* 0.01). There were no significant differences in the expression levels of *HEMD* (encoding uroporphyrinogen III synthase), *CHLG* (encoding Chl synthase), *CHLM* (encoding Mg-protoporphyrin IX methyltransferase), *HEMG2* (encoding protoporphyrinogen III oxidase), or *CHLI* (encoding Mg-chelatase I subunit).

**Fig. 5:**
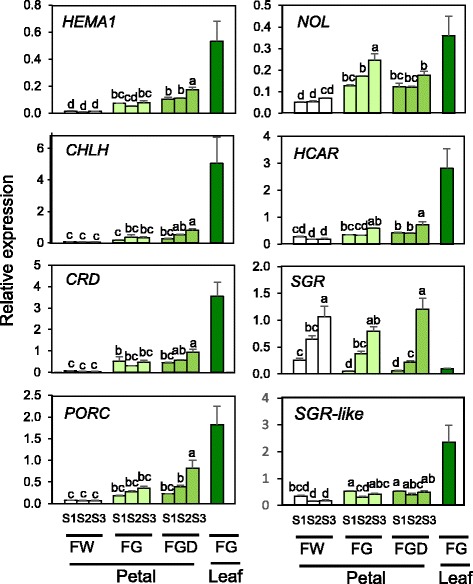
RT-qPCR analysis of Chl metabolic genes in FW, FG, and FGD. Mean values (± SD) of three biological replicates are shown. Designations of petal developmental stages and cultivars are as in Fig. 2. Gene abbreviations are as in Fig. 1. The differences among petals (FW, FG, and FGD) were analyzed by Tukey–Kramer multiple-comparison test. Different letters indicate significant differences at *P* < 0.05

Because the expression patterns of group 1 genes were positively matched with Chl content, we hypothesized that these genes play an important role in determining Chl content in petals and further analyzed their expression by RT-qPCR. As in microarray analysis, the levels of *CHLH*, *CRD*, *HEMA1*, and *PORC* transcripts were highest in leaves and were extremely low in FW petals (Fig. 5). In FGD, the expression of all four genes tended to increase as petals matured.

#### Chl cycle

Among genes related to the Chl cycle, the expression of two group 2 genes, *NOL* (*Non-yellow coloring one-like*, encoding Chl *b* reductase subunit) and *HCAR* (encoding hydroxymethyl Chl *a* reductase), was well correlated with Chl content: it was lower in FW petals than in FG and FGD petals and was remarkably high in FG leaves (Fig. 4b). RT-qPCR analysis of *NOL* and *HCAR* showed similar expression patterns to those observed in the microarray analysis (Fig. 5). In FG and FGD, the expression of group 2 genes tended to increase as petals matured.

#### Chl degradation

Among genes related to Chl degradation, the expression of only *SGR* (*STAY-GREEN*, encoding Mg-dechelatase) showed negative correlation with Chl content, with the highest expression in FW petals and extremely low in leaves (Fig. 4c). There was no clear relationship between Chl content and expression levels of *RCCR* (encoding red Chl catabolite reductase), *PaO* (encoding pheophorbide *a* oxygenase), and *PPH* (encoding pheophytinase). RT-qPCR analysis showed that the expression levels of *SGR* drastically increased as petals matured in FW, FG, and FGD, whereas that in leaves was very low (Fig. 5). Because an oligo probe specific to the *SGR-like* gene, a homologue of *SGR*, was absent from our custom oligonucleotide array, *SGR-like*–specific primers were designed based on its chrysanthemum EST sequence, and RT-qPCR was performed. Unlike *SGR* expression, that of *SGR-like* was lower in petals than in leaves during the course of development.

### Comparison of gene expression between green and white petals

**Fig. 6:**
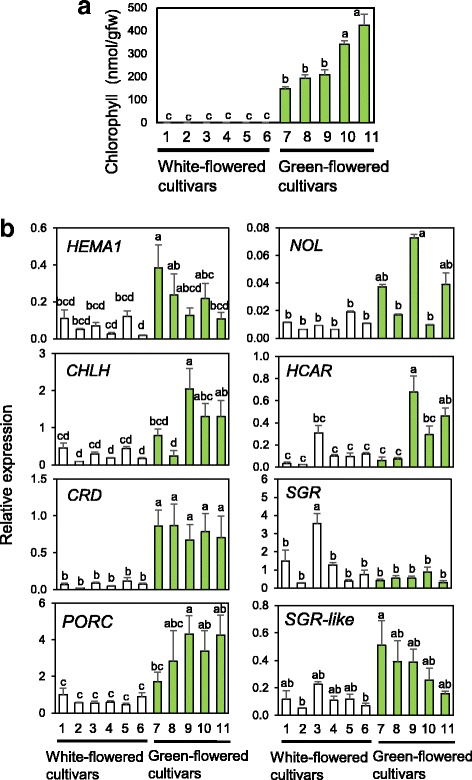
RT-qPCR analysis of Chl metabolic genes in petals of white- and green-flowered chrysanthemum cultivars. **a** Chl content; (**b**) expression of selected genes related to Chl metabolism. Mean values (± SD) of three biological replicates are shown. Different letters indicate significant differences by Tukey–Kramer multiple-comparison test (*P* < 0.05). Gene abbreviations are as in Fig. 1. White-flowered cultivars: 1, Estrella; 2, Baltica White; 3, Ping Pong Super; 4, Sei Elsa; 5, Radost; 6, Ferry. Green-flowered cultivars: 7, Sei Green Needle; 8, Greea; 9, Anastasia Green; 10, Olive; 11, Green Lizard

To confirm our findings, we compared the expression of group 1 and 2 genes and *SGR* (Fig. 4) in S3 petals of several white- and green-flowered cultivars (Additional file 4 Figure S2). Chl content in S3 petals was <1.4 nmol/g fresh weight (g FW) in white-flowered cultivars and 147–424 nmol/g FW in green-flowered cultivars (Fig. 6a). Among group 1 genes (*CRD*, *CHLH*, *HEMA1*, and *PORC*), only the expression of *CRD* was significantly higher in green petals than in white petals in all cultivars tested (Fig. 6b). Expression of the other three genes tended to be higher in green petals than in white petals in most cultivars compared. There was a considerable variability among cultivars in the expression levels of group 2 genes (*NOL* and *HCAR*) and *SGR*, with no significant correlation between transcript levels and Chl content.

### Transcription factors differentially expressed in FW and FGD petals in comparison with FG petals

To identify transcription factors involved in the regulation of Chl accumulation in petals, we analyzed transcription factor genes whose expression was positively or negatively related to Chl content. Tables 1 and 2 list transcription factor genes differentially expressed between FG and FW petals and between FG and FGD petals, respectively, in our microarray data (*P* < 0.01 and fold change > ±15).

**Table 1:** Transcription factors differentialy expressed between FG and FW petals (fold change >15 or > −15)

Probe ID		GenBank accession no	Fold Change	*P*-Value	Closest Arabidopsis gene Classification		Arabidopsis annotation
PD0028_011900	*	IABW01012977	84.8	8.E-07	AT3G52525.1	OFP	*Arabidopsis thaliana* OVATE FAMILY PROTEIN 6, OFP6 O
PD0028_044537	*	IABW01054620	60.4	9.E-05	AT1G66370.1	MYB	myb domain protein 113
PD0028_148815		IABW01198808	50.1	1.E-04	AT1G66370.1	MYB	myb domain protein 113
PD0028_075950		IABW01104970	48.9	1.E-05	AT1G66370.1	MYB	myb domain protein 113
PD0028_154888		IABW01206257	32.4	3.E-04	AT2G36026.1	OFP	Ovate family protein
PD0028_150392		IABW01200686	31.7	3.E-05	AT3G52525.1	OFP	ARABIDOPSIS THALIANA OVATE FAMILY PROTEIN 6, OFP6 O
PD0028_033761		IABW01039606	30.7	2.E-05	AT1G59750.1	ARF	auxin response factor 1
PD0028_044920		IABW01055129	28.0	4.E-04	AT1G66370.1	MYB	myb domain protein 113
PD0028_048158		IABW01059862	25.8	1.E-04	AT1G19510.1	MYB	RADIALIS-LIKE SANT/MYB 4, RAD-like 5
PD0028_081008		IABW01114181	25.6	2.E-04	AT1G60700.1	FHA	SMAD/FHA domain-containing protein
PD0028_103797		IABW01145611	15.3	7.E-03	AT4G17880.1	bHLH	Basic helix-loop-helix (bHLH) DNA-binding family protein
PD0028_168726		IABW01092014	−296.7	1.E-03	AT5G06839.3	bZIP	TGACG (TGA) motif-binding protein 10
PD0028_094749		IABW01134810	−38.8	0.E + 00	AT5G67190.1	AP2_ERF	DREB and EAR motif protein 2
PD0028_062703		IABW01082439	−36.4	0.E + 00	AT5G67190.1	AP2_ERF	DREB and EAR motif protein 2
PD0028_043748		IABW01053524	−36.0	0.E + 00	AT5G67190.1	AP2_ERF	DREB and EAR motif protein 2
PD0028_010170		IABW01011053	−35.0	0.E + 00	AT1G75250.1	MYB	RADIALIS-LIKE SANT/MYB 3, RAD-like 6
PD0028_048604	*	IABW01060484	−33.6	0.E + 00	AT5G67190.1	AP2_ERF	DREB and EAR motif protein 2
PD0028_069119		IABW01093153	−30.6	2.E-03	AT4G36740.1	HD	homeobox protein 40, HD40
PD0028_076607	*	IABW01106213	−29.3	3.E-03	AT4G36740.1	HD	homeobox protein 40, HD40
PD0028_046802		IABW01058023	−28.9	2.E-03	AT4G36740.1	HD	homeobox protein 40, HD40
PD0028_093450		IABW01133272	−25.8	1.E-03	AT4G36740.1	HD	homeobox protein 40, HD40
PD0028_036988		IABW01044039	−24.4	1.E-03	AT4G36740.1	HD	homeobox protein 40, HD40
PD0028_003905	*	IABW01004158	−22.6	0.E + 00	AT1G25440.1	CO	B-box type zinc finger protein with CCT domain, COL16
PD0028_009958		IABW01010820	−20.7	1.E-03	AT1G75410.1	HD	BEL1-like homeodomain 3
PD0028_150651		IABW01201015	−18.4	2.E-03	AT5G52020.1	AP2_ERF	Integrase-type DNA-binding superfamily protein
PD0028_029276		IABW01034060	−18.4	0.E + 00	AT1G68520.1	CO	B-box type zinc finger protein with CCT domain, COL6
PD0028_001574		IABW01001664	−18.3	1.E-03	AT5G14280.1	GeBP	DNA-binding storekeeper protein-related
PD0028_100279		IABW01141429	−18.2	1.E-03	AT3G11580.1	B3	AP2/B3-like transcriptional factor family protein
PD0028_107134		IABW01149631	−17.7	0.E + 00	AT5G65050.3	MADS	MADS AFFECTING FLOWERING 2, AGAMOUS-like 31
PD0028_074156		IABW01101743	−17.6	1.E-03	AT2G43060.1	bHLH	ILI1 binding bHLH 1
PD0028_082618		IABW01117162	−16.6	4.E-03	AT5G52020.1	AP2_ERF	Integrase-type DNA-binding superfamily protein
PD0028_145445		IABW01194763	−16.1	0.E + 00	AT5G56860.1	GATA	GATA, nitrate-inducible, carbon metabolism-involved
PD0028_082089		IABW01116183	−15.9	3.E-03	AT5G52020.1	AP2_ERF	Integrase-type DNA-binding superfamily protein
PD0028_098862		IABW01139756	−15.8	3.E-03	AT5G52020.1	AP2_ERF	Integrase-type DNA-binding superfamily protein
PD0028_069340		IABW01093503	−15.8	2.E-03	AT5G52020.1	AP2_ERF	Integrase-type DNA-binding superfamily protein
PD0028_117926		IABW01162197	−15.8	5.E-03	AT1G25440.1	CO	B-box type zinc finger protein with CCT domain, COL16
PD0028_148039		IABW01197925	−15.8	1.E-03	AT3G23250.1	MYB	myb domain protein 15
PD0028_129536		IABW01175867	−15.4	1.E-03	AT1G32150.1	bZIP	basic region/leucine zipper transcription factor 68

**Fig. 7:**
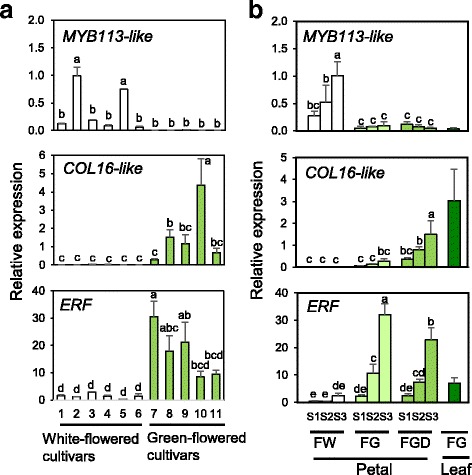
RT-qPCR analysis of transcription factor genes differentially expressed in petals of white- and green-flowered chrysanthemum cultivars. Mean values (± SD) of three biological replicates are shown. Different letters indicate significant differences by Tukey–Kramer multiple-comparison test (*P* < 0.05). Designations of petal developmental stages and cultivars are as in Figs. 2 and 6

* Genes whose expression levels in the white and green petals were analyzed by RT-qPCR (Figs.6, 7, and S3)

**Table 2:** Transcription factors differentialy expressed between FG and FGD petals (fold change >15 or > −15)

Probe ID		GenBank accession no.	Fold Change	*P*-Value	Closest Arabidopsis gene	Classification	Arabidopsis annotation
PD0028_006794		IABW01007316	100.5	6.E-04	AT1G75250.1	MYB	RADIALIS-LIKE SANT/MYB 3, RAD-like 6
PD0028_092972	*	IABW01132689	91.4	9.E-04	AT1G75250.1	MYB	RADIALIS-LIKE SANT/MYB 3, RAD-like 6
PD0028_048158		IABW01059862	42.8	2.E-04	AT1G19510.1	MYB	RADIALIS-LIKE SANT/MYB 4, RAD-like 5
PD0028_034984	*	IABW01041136	29.7	2.E-03	AT3G17730.1	NAC	NAC domain containing protein 57
PD0028_033512	*	IABW01039301	25.2	6.E-03	AT2G27300.1	NAC	Arabidopsis NAC domain containing protein 40, NTM1-like 8, NTL8
PD0028_007777		IABW01008418	23.5	3.E-02	AT4G32280.1	AUX_IAA	indole-3-acetic acid inducible 29
PD0028_190240		IABW01206912	21.6	1.E-05	AT5G05120.1	C2H2ZnF	C2H2 and C2HC zinc fingers superfamily protein
PD0028_071210	*	IABW01096525	20.4	4.E-03	AT5G25390.2	AP2_ERF	shine3, SHN3, Integrase-type DNA-binding superfamily protein
PD0028_064158		IABW01084746	19.8	6.E-04	AT1G19510.1	MYB	RADIALIS-LIKE SANT/MYB 4, RAD-like 5
PD0028_102746	*	IABW01144347	19.3	5.E-04	AT1G19510.1	MYB	RADIALIS-LIKE SANT/MYB 4, RAD-like 5
PD0028_113111		IABW01156549	18.4	3.E-03	AT5G42630.1	GARP	KANADI 4, ABERRANT TESTA SHAPE
PD0028_037235		IABW01044426	18.0	4.E-03	AT5G25390.2	AP2_ERF	shine3, SHN3, Integrase-type DNA-binding superfamily protein
PD0028_081008		IABW01114181	17.9	4.E-04	AT1G60700.1	FHA	SMAD/FHA domain-containing protein
PD0028_142351		IABW01190973	15.0	3.E-04	AT3G62240.1	C2H2ZnF	RING/U-box superfamily protein
PD0028_132595		IABW01179425	−179.5	2.E-05	AT3G28917.1	ZF_HD	mini zinc finger 2, MIF2
PD0028_125957		IABW01171648	−142.3	2.E-05	AT3G28917.1	ZF_HD	mini zinc finger 2, MIF2
PD0028_105609		IABW01147785	−138.9	4.E-05	AT3G28917.1	ZF_HD	mini zinc finger 2, MIF2
PD0028_043321		IABW01052932	−122.2	4.E-05	AT3G28917.1	ZF_HD	mini zinc finger 2, MIF2
PD0028_064889		IABW01085974	−96.2	2.E-05	AT3G28917.1	ZF_HD	mini zinc finger 2, MIF2
PD0028_041425	*	IABW01050224	−68.9	6.E-05	AT3G28917.1	ZF_HD	mini zinc finger 2, MIF2
PD0028_076231		IABW01105570	−20.0	7.E-03	AT2G37060.1	CCAAT	nuclear factor Y, subunit B8, nuclear factor Y, subunit B8
PD0028_086808		IABW01124961	−18.9	3.E-03	AT5G51990.1	AP2_ERF	DEHYDRATION-RESPONSIVE ELEMENT-BINDING PROTEIN 1D
PD0028_042367		IABW01051507	−17.9	2.E-03	AT4G34410.1	AP2_ERF	redox responsive transcription factor 1
PD0028_148039		IABW01197925	−15.6	3.E-04	AT3G23250.1	MYB	myb domain protein 15

* Genes whose expression levels in the white and green petals were analyzed by RT-qPCR (Figs.6, 7, and S3)

We identified 11 transcription factor genes expressed significantly higher in FW petals than in FG petals (Table 1, upper part). Five of them encoded MYB domain proteins and showed high fold change values (25.8–60.4). Four of these *MYB* sequences were most closely related to *MYB113* in the Arabidopsis R2R3-Myb family and were designated *MYB113-like*. Arabidopsis MYB113 is involved in flavonoid biosynthesis [18, 19]. However, the expression of one of the *MYB113-like* genes was not associated with that of the flavonoid biosynthesis genes dihydroflavonol 4-reductase, chalcone synthase, anthocyanidin synthase, and chalcone isomerase (Additional file 5 Figure S3). A gene encoding an ovate family protein showed the highest fold change value of 84.8. Expression of 27 transcription factor genes was down-regulated in FW petals, including 4 DREB and EAR motif protein (ERF) genes, 5 homeobox protein 40 (HD40) genes, 5 integrase-type DNA-binding superfamily protein genes, and 3 B-box type zinc finger protein with CCT domain (CONSTANS-like, COL) genes (Table 1, lower part). The *COL* gene sequences were most closely related to *COL6* or *COL16* in Arabidopsis and were designated *COL16-like*.

We identified 14 transcription factor genes expressed significantly higher in FGD petals than in FG petals (Table 2, upper part). Among them, two genes encoding MYB3 proteins showed the highest fold change values (100.5 and 91.4). Three genes encoding MYB4 also showed high fold change values (19.3–42.8). Ten transcription factor genes were expressed at significantly lower levels in FGD petals than in FG petals (Table 2, lower part). Six of them encoded mini zinc finger 2 proteins and showed extremely low relative expression in FGD petals (fold change values from −68.9 to −179.5).

To validate petal color–specific expression, we performed RT-qPCR analysis of 11 transcription factor genes in S3 petals of white- and green-flowered chrysanthemum cultivars. These genes were selected as representatives of different groups of transcription factors identified by the microarray analysis (Tables 1 and 2). Among them, the expression of *MYB113-like* was restricted to white petals (Fig. 7a), although there was a variability in the expression levels among cultivars. In contrast, the expression of *ERF* and *COL16-like* tended to be higher or was significantly higher in green petals than in white petals (Fig. 7a). There was no correlation between the transcript levels of the other 8 genes and Chl content (Additional file 6 Figure S4).

We also analyzed the expression of *MYB113-like*, *COL16-like*, and *ERF* during development of FW, FG, and FGD petals, and in FG leaves. The expression of a *MYB113-like* gene in FW increased as petals matured, whereas it remained low in FG and FGD petals, and was also low in leaves (Fig. 7b). The expression of a *COL16-like* gene was extremely low in FW petals and high in FG leaves. FGD petals showed higher *COL16-like* gene expression than did FW and FG petals, and the expression increased as FG and FGD petals matured. The expression of an *ERF* gene was highest in FG and FGD petals and increased as petals matured.

We also examined the expression profiles of transcription factors previously identified as negative or positive regulators of Chl biosynthesis. GLK, HY5, and GNC enhance Chl biosynthesis [7–9]. ESTs encoding these transcription factors showed different expression patterns: *GNC* and *HY5* expression in petals was positively and negatively correlated with Chl content, respectively. There was no marked difference in the expression level of *GLK* in petals of FW, FG and FGD. PIF1 and PIF3, have been shown to negatively regulate Chl biosynthesis [10]. However, in our microarray analysis, *PIF1* and *PIF3* expression levels were positively associated with Chl content in FW, FG, and FGD petals (Additional file 7 Figure S5). The results suggest that these transcription factors are not involved in the regulation of Chl accumulation in petals and that different transcription factors control Chl accumulation in leaves and petals.

### Comparison of plastid ultrastructure in FW, FG, and FGD petals

**Fig. 8:**
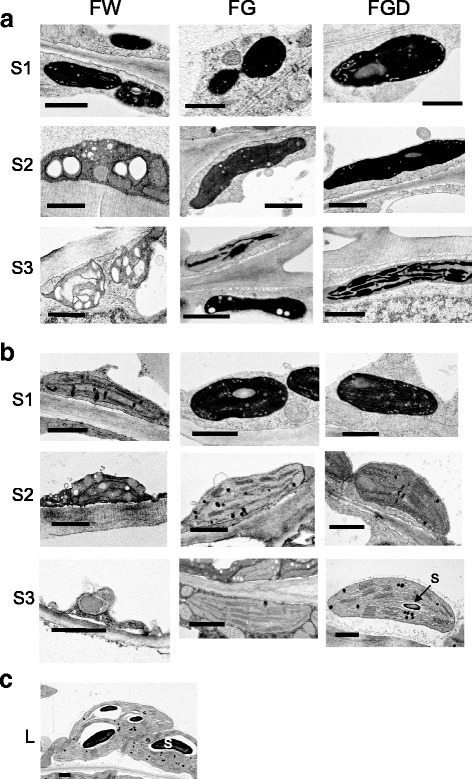
Transmission electron microscopy of plastid ultrastructure. Plastids from (**a**) epidermal cells and (**b**) mesophyll cells of FW, FG, and FGD petals at different stages. (**c**) Plastids from a mature FG leaf. Bar = 1 μm. S, starch granule

We compared plastid ultrastructure among FW, FG, and FGD petals at different developmental stages and FG leaves by using TEM. At S1, epidermal cells of all petals contained electron-dense small plastids (Fig. 8a). Mesophyll cells of all petals also contained small plastids with lower electron density (Fig. 8b). At S2, the development of membrane structures was evident in the plastids of mesophyll cells of FG and FGD petals, whereas those of FW cells contained plastids with destroyed thylakoid membranes (Fig. 8b). At S3, we observed plastid debris in both epidermal and mesophyll cells of FW petals (Fig. 8a and b; Additional file 8 Figure S6). In contrast, S3 FG and FGD petals still contained plastids with a well-developed thylakoid system. In leaves, plastids were larger than those in FG and FGD petals at S3 and contained large starch granules (Fig. 8c). Plastids were found only in mesophyll but not in epidermal cells (Additional file 8 Figure S6).

## Discussion

To clarify the key factors that determine different levels of Chl accumulation in leaves and petals of chrysanthemums, we performed microarray analysis and searched for the differences in the expression levels of genes. GO classification showed that, among the downregulated genes in FW petals, genes associated with “plastids”, “chloroplasts”, and “electron transport or energy pathways” were highly represented. These results indicate that many of the genes related to photosynthesis whose products function in the chloroplast were transcriptionally inactive in FW petals. TEM observation of petals supports the hypothesis: at the late stage of development, green petals contain chloroplasts with well-developed thylakoid membranes, whereas white petals have lost chloroplasts. TEM observation also showed that, in green petals, both mesophyll and epidermal cells contain plastids, whereas only mesophyll cells contain plastids in leaves. The result indicates that leaves and petals have different patterns of Chl accumulation: Chls are present only in mesophyll cells in leaves, whereas they are present in both epidermal and mesophyll cells in green petals. Therefore, it is reasonable to assume that the regulatory mechanisms for Chl accumulation differ in leaves and petals.

Microarray analysis showed that the expression of many Chl biosynthesis genes was lower in white petals than in green petals. In particular, the expression of genes encoding CHLH, CRD, HEMA1, and PORC was well associated with Chl content in petals and leaves of the FW, FG, and FGD cultivars. It is worth noting that the expression of the genes encoding these enzymes is induced by light and they are considered as key enzymes for Chl biosynthesis during photomorphogenesis [20–22]. We propose that Chl biosynthesis activity is lower in white petals than in leaves and green petals because of the remarkably low levels of expression of these genes.

There was no association between the expression levels of most Chl catabolic genes and Chl content in the petals of FW, FG, and FGD. However, *SGR* expression in mature petals of these cultivars was markedly higher than that in leaves. SGR is responsible for stay-green phenotypes of leaves in rice and Arabidopsis [23, 24] and also non-photosynthetic tissues such as fruits of tomato, pepper, and kiwi, in which Chl degradation normally occurs at the onset of fruit ripening [25, 26]. Several lines of evidence indicate that SGR plays a key role in the initiation of Chl degradation by destabilizing protein–pigment complexes in the thylakoid membranes [27, 28]. Very recently, Shimoda et al. [29] revealed that SGR is a Mg-dechelatase, which catalyzes the conversion of Chl *a* to pheophytin *a*, which is the first step of Chl degradation. We then assume that SGR maintains high Chl catabolic activity and contributes to the absence or low level of Chls in chrysanthemum petals. Our results on the expression patterns of Chl metabolic genes in chrysanthemums are similar to our previous results on carnation and Arabidopsis [6].

Based on our results, we hypothesize that a low rate of Chl biosynthesis and a high rate of Chl degradation lead to the absence of Chls in white chrysanthemum petals. We assumed that factors that suppress Chl biosynthesis may exist in non-green petals. Higher rate of Chl biosynthesis in green petals may result from a loss or reduced activity of the suppressors. We then searched for transcription factors whose expression is related to Chl content and identified *COL16-like*, *ERF*, and *MYB113-like*, which were coordinately expressed with Chl content in petals.

The white petal–specific *MYB113-like* genes belong to the R2R3-MYB family, which controls plant-specific physiological processes including primary and secondary metabolism, cell fate and identity, development, and responses to biotic and abiotic stresses [30]. Arabidopsis MYB113 belongs to subclass 6, which is involved in flavonoid biosynthesis [18, 19], but the expression level of the chrysanthemum *MYB113-like* gene in petals was not associated with those of flavonoid biosynthesis genes (Additional file 5 Figure S3). We showed that a *COL16-like* gene was highly expressed in green petals and leaves of chrysanthemum. In Arabidopsis and *Medicago truncatula*, the COL protein family is divided into Groups I to III on the basis of conserved domains [31]. Group I might regulate flowering time [32–34]. The EST sequence of chrysanthemum *COL16-like* was most similar to Arabidopsis *AtCOL16* (AT1G25440) of Group II, for which limited information about their physiological function is available [35]. Another green petal–specific transcription factor, ERF, belongs to the APETALA2 (AP2) family. ERFs control growth and development as well as responses to environmental stimuli [36, 37]. Several ERFs regulate primary and secondary metabolism. For example, *Catharanthus roseus* ERFs (ORCA2 and ORCA3) enhance the jasmonate-responsive expression of strictosidine synthase, which is required for terpenoid indole alkaloid synthesis [38]. To our knowledge, the involvement of COL16 and ERF in Chl metabolism has never been reported.

## Conclusion

From our transcriptome analysis, we suggest that the low expression levels of chlorophyll biosynthesis genes and the high expression levels of a chlorophyll catabolic gene lead to the absence of chlorophylls in white chrysanthemum petals. We hypothesize that factor(s) that suppress(es) chlorophyll biosynthesis gene expression may exist in white petals. Higher rate of chlorophyll biosynthesis in green petals may result from the loss of such suppressors. We identified candidate transcriptional regulators that may be involved in chlorophyll metabolism and/or accumulation in petals. To provide further insight into the regulatory mechanism of tissue-specific chlorophyll accumulation, the function of the candidate transcription factors should be evaluated.

## Additional files

Additional file 1: Table S1. Primers used for RT-qPCR analysis. (PPTX 67 kb)

Additional file 2: Figure S1. Chlorophyll content in FW, FG, and FGD leaves. Mean values (± SD) of three biological replicates are shown. (PPTX 45 kb)

Additional file 3: Table S2. List of chlorophyll metabolic genes. (PPTX 69 kb)

Additional file 4: Figure S2. Photographs of flowers of white- and green-flowered chrysanthemum cultivars used for RT-qPCR analysis presented in Figs. 6 and 7, and Additional file 5 **Figure S4.** (PPTX 1046 kb)

Additional file 5: Figure S3. Expression of *MYB113-like* and anthocyanin biosynthesis genes in FW, FG, and FGD. Microarray data were obtained as described in Fig. 4. *ANS*, anthocyanidin synthase; *CHI*, chalcone isomerase; *CHS*, chalcone synthase; *DFR*, dihydroflavonol 4-reductase. GenBank accession number of each gene is indicated in parentheses. (PPTX 50 kb)

Additional file 6: Figure S4. RT-qPCR analysis of selected transcription factor genes in petals of white- and green-flowered chrysanthemum cultivars. Cultivar numbers are as in Additional file 4 **Figure S2.** A total of 11 genes were selected by microarray analysis (Tables 1 and 2). Three of them were differentially expressed between white and green petals (Fig. 7). The expression levels of the remaining 8 genes (presented in this **Figure S4.**), were not significantly different between white and green petals. Different letters indicate significant differences in Tukey–Kramer multiple-comparison test (*P* < 0.05). (PPTX 108 kb)

Additional file 7: Figure S5. Expression of Chl-related transcription factor genes in FW, FG, and FGD. Microarray data were obtained as described in Fig. 4. The GenBank accession number of each gene is indicated in parentheses. (PPTX 49 kb)

Additional file 8: Figure S6. Transmission electron microscopy at low magnification. Transverse sections of petals at stages 1 to 3 (S1 to S3), and leaves (L). The uppermost cell layer in each photograph is the epidermal cell layer. Bar = 20 μm. (PPTX 10712 kb)

## Abbreviations

CGA1: Cytokinin responsive gata factor1
Chl: Chlorophyll
CHLG: Chl synthase
CHLH: Mg-protoporphyrin IX chelatase (Mg-chelatase) H subunit
CHLI: Mg-chelatase I subunit
CHLM: Mg-protoporphyrin IX methyltransferase
COL: CONSTANS-like
CRD: Mg-protoporphyrin IX monomethylester cyclase
DVR: Divinyl chlorophyllide *a* 8-vinyl-reductase
ERF: DREB and EAR motif protein
EST: Expressed sequence tag
GLK: GOLDEN2-LIKE
GNC: Gata, nitrate-inducible, carbon-metabolism involved
GNL: GNC-LIKE
GO: Gene ontology
HCAR: Hydroxymethyl Chl *a* reductase
HEMA: Glutamyl-tRNA reductase
HEMB: 5-aminolevulinate dehydratase
HEMD: Uroporphyrinogen III synthase
HEMG: Protoporphyrinogen III oxidase
HY5: Long hypocotyl5
NOL: Non-yellow coloring one-like
PaO: Pheophorbide *a* oxygenase
PIF: Phytochrome-interacting factor
PORC: Protochlorophyllide oxidoreductase C
PPH: Pheophytinase
RCCR: Red Chl catabolite reductase
RT-qPCR: Quantitative real-time PCR
SGR: Stay-green
TEM: Transmission electron microscopy
