# Supplementary material for: Transcriptome analysis in petals and leaves of chrysanthemums with different chlorophyll levels
Source: BMC Plant Biol. 2017 Nov 15;17:202. doi: 10.1186/s12870-017-1156-6 (PMC5688696; doi:10.1186/s12870-017-1156-6)
Supplement: Supplementary file 1 — Primers used for RT-qPCR analysis. (PPTX 67 kb) [file 12870_2017_1156_MOESM1_ESM.pptx]

## Slide 1
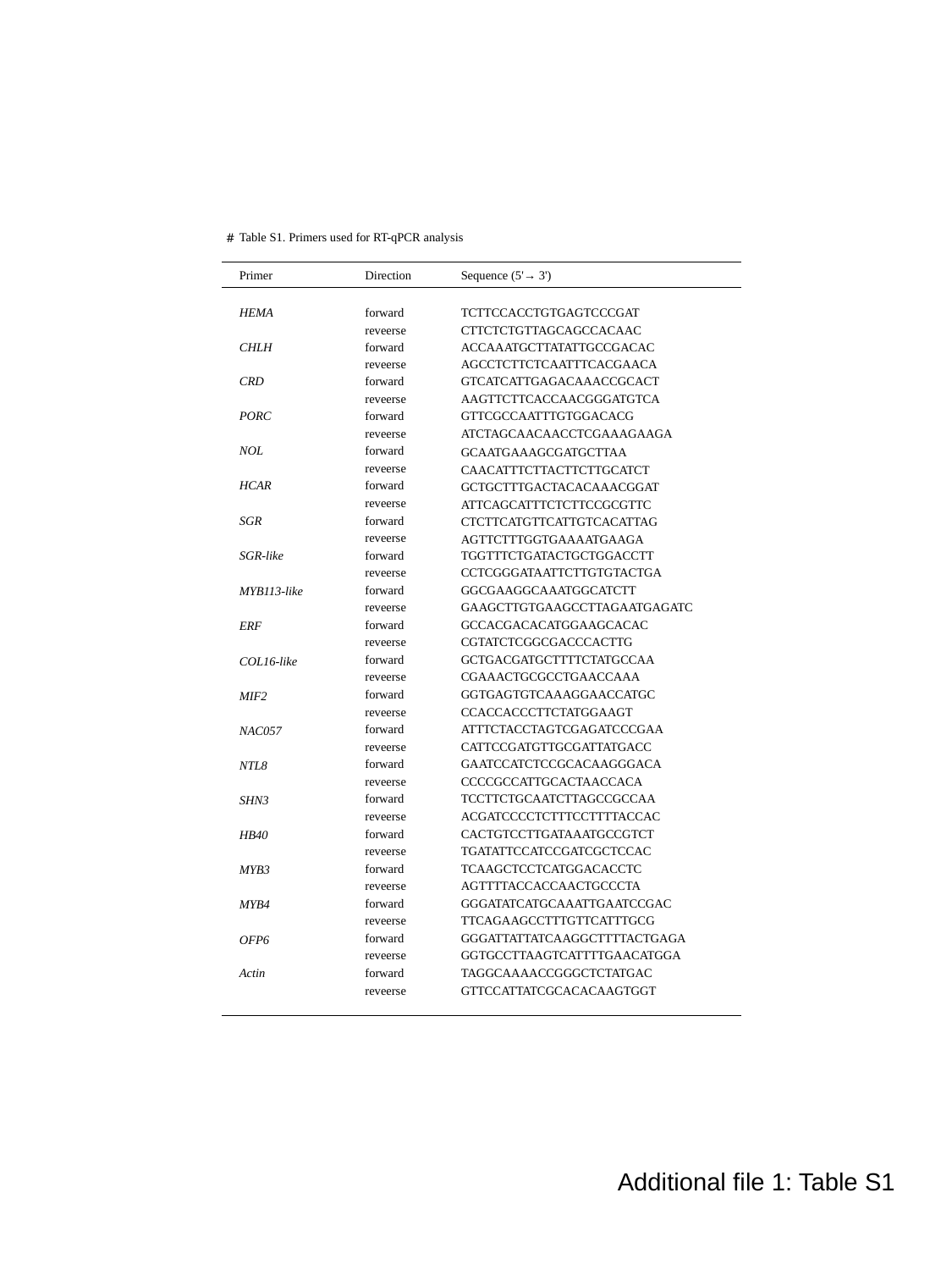

| # | Table S1. Primers used for RT-qPCR analysis | | |
| --- | --- | --- | --- |
| | | | |
| | Primer | Direction | Sequence (5'→ 3') |
| | | | |
| | HEMA | forward | TCTTCCACCTGTGAGTCCCGAT |
| | | reveerse | CTTCTCTGTTAGCAGCCACAAC |
| | CHLH | forward | ACCAAATGCTTATATTGCCGACAC |
| | | reveerse | AGCCTCTTCTCAATTTCACGAACA |
| | CRD | forward | GTCATCATTGAGACAAACCGCACT |
| | | reveerse | AAGTTCTTCACCAACGGGATGTCA |
| | PORC | forward | GTTCGCCAATTTGTGGACACG |
| | | reveerse | ATCTAGCAACAACCTCGAAAGAAGA |
| | NOL | forward | GCAATGAAAGCGATGCTTAA |
| | | reveerse | CAACATTTCTTACTTCTTGCATCT |
| | HCAR | forward | GCTGCTTTGACTACACAAACGGAT |
| | | reveerse | ATTCAGCATTTCTCTTCCGCGTTC |
| | SGR | forward | CTCTTCATGTTCATTGTCACATTAG |
| | | reveerse | AGTTCTTTGGTGAAAATGAAGA |
| | SGR-like | forward | TGGTTTCTGATACTGCTGGACCTT |
| | | reveerse | CCTCGGGATAATTCTTGTGTACTGA |
| | MYB113-like | forward | GGCGAAGGCAAATGGCATCTT |
| | | reveerse | GAAGCTTGTGAAGCCTTAGAATGAGATC |
| | ERF | forward | GCCACGACACATGGAAGCACAC |
| | | reveerse | CGTATCTCGGCGACCCACTTG |
| | COL16-like | forward | GCTGACGATGCTTTTCTATGCCAA |
| | | reveerse | CGAAACTGCGCCTGAACCAAA |
| | MIF2 | forward | GGTGAGTGTCAAAGGAACCATGC |
| | | reveerse | CCACCACCCTTCTATGGAAGT |
| | NAC057 | forward | ATTTCTACCTAGTCGAGATCCCGAA |
| | | reveerse | CATTCCGATGTTGCGATTATGACC |
| | NTL8 | forward | GAATCCATCTCCGCACAAGGGACA |
| | | reveerse | CCCCGCCATTGCACTAACCACA |
| | SHN3 | forward | TCCTTCTGCAATCTTAGCCGCCAA |
| | | reveerse | ACGATCCCCTCTTTCCTTTTACCAC |
| | HB40 | forward | CACTGTCCTTGATAAATGCCGTCT |
| | | reveerse | TGATATTCCATCCGATCGCTCCAC |
| | MYB3 | forward | TCAAGCTCCTCATGGACACCTC |
| | | reveerse | AGTTTTACCACCAACTGCCCTA |
| | MYB4 | forward | GGGATATCATGCAAATTGAATCCGAC |
| | | reveerse | TTCAGAAGCCTTTGTTCATTTGCG |
| | OFP6 | forward | GGGATTATTATCAAGGCTTTTACTGAGA |
| | | reveerse | GGTGCCTTAAGTCATTTTGAACATGGA |
| | Actin | forward | TAGGCAAAACCGGGCTCTATGAC |
| | | reveerse | GTTCCATTATCGCACACAAGTGGT |
| | | | |
| | | | |
Additional file 1: Table S1
