# Supplementary figures and images for: Transcriptome analysis in petals and leaves of chrysanthemums with different chlorophyll levels
Source: BMC Plant Biol. 2017 Nov 15;17:202. doi: 10.1186/s12870-017-1156-6 (PMC5688696; doi:10.1186/s12870-017-1156-6)

## Slide 1
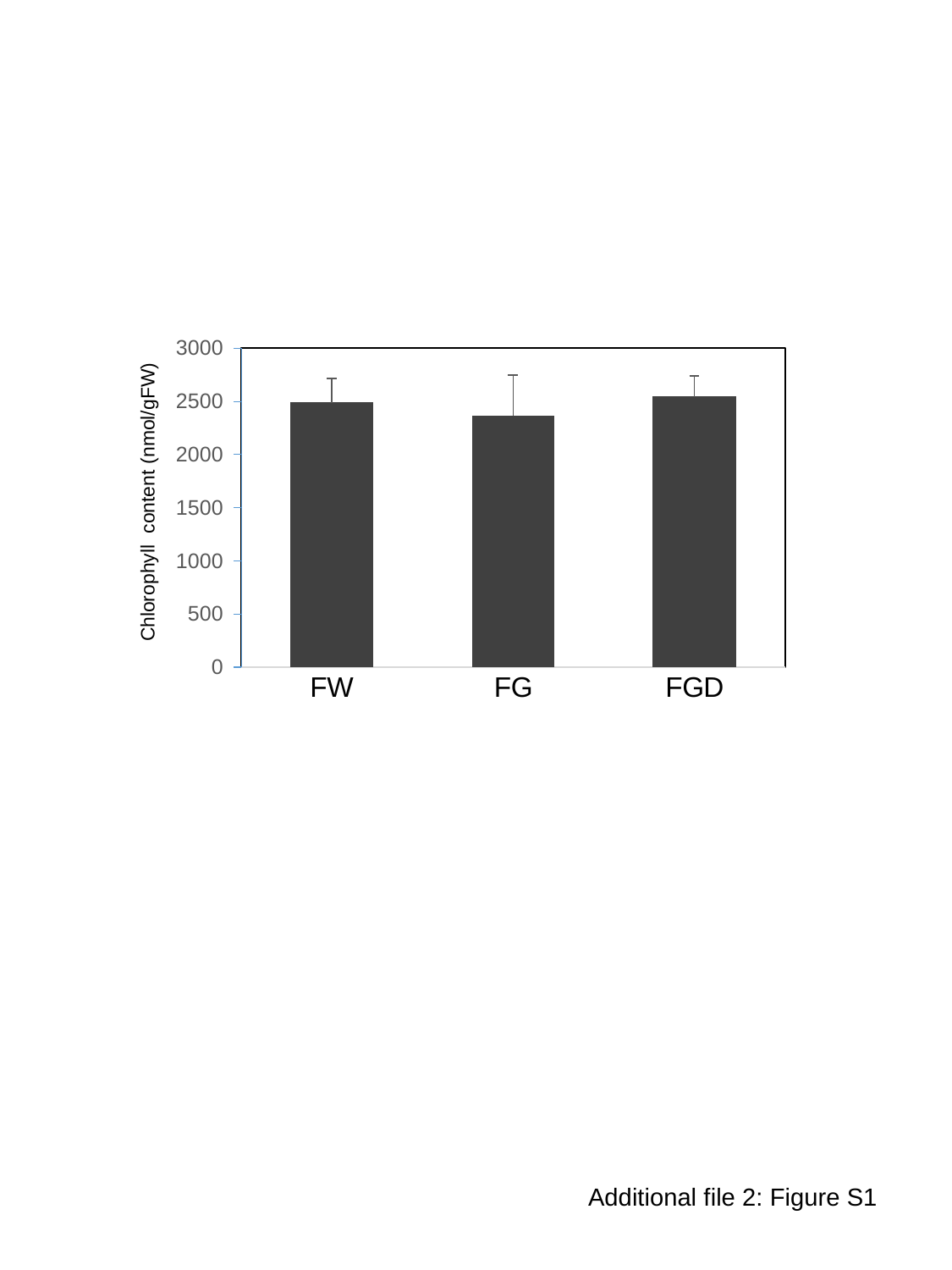

### Chart
| Category | |
|---|---|
| FW | 2488.0 |
| FG | 2362.5046985059676 |
| FGD | 2542.0 |Chlorophyll content (nmol/gFW)
Additional file 2: Figure S1

Supplement: Supplementary file 2 — Chlorophyll content in FW, FG, and FGD leaves. Mean values (± SD) of three biological replicates are shown. (PPTX 45 kb) [file 12870_2017_1156_MOESM2_ESM.pptx]

## Slide 1
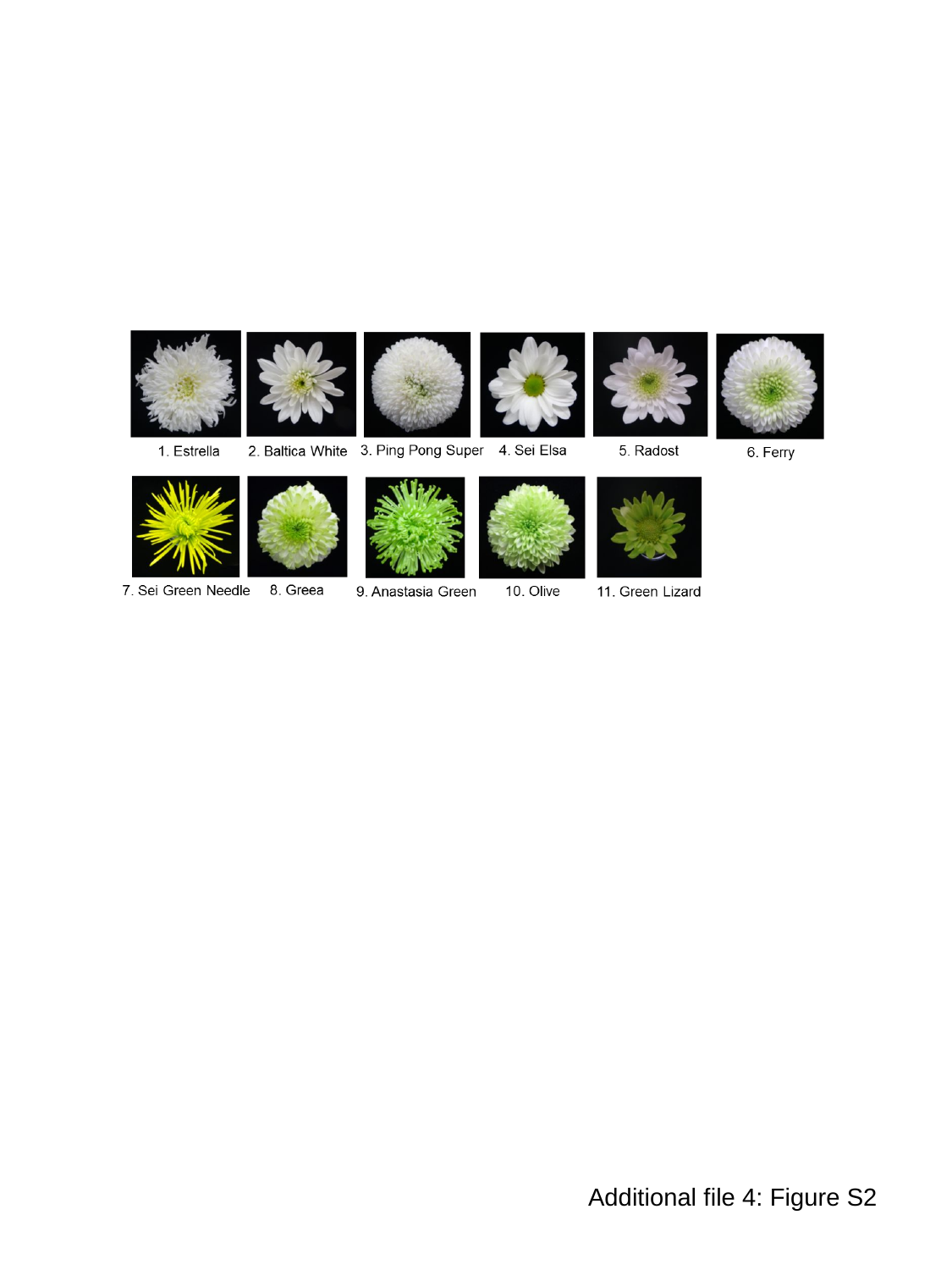

Additional file 4: Figure S2

Supplement: Supplementary file 4 — Photographs of flowers of white- and green-flowered chrysanthemum cultivars used for RT-qPCR analysis presented in Figs. 6 and 7, and Additional file 5 Figure S4. (PPTX 1046 kb) [file 12870_2017_1156_MOESM4_ESM.pptx]

## Slide 1
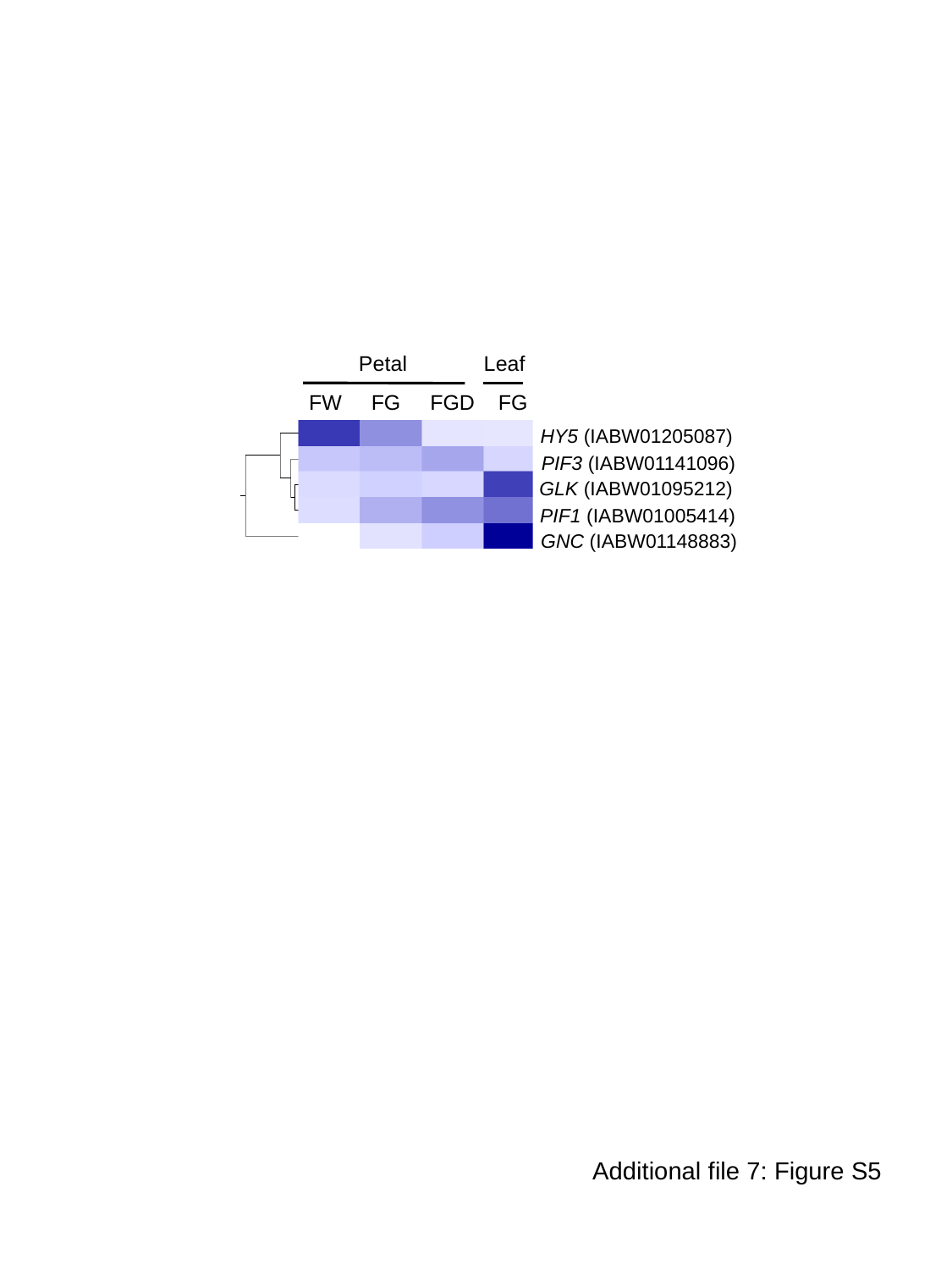

Petal Leaf
FW FG FGD FG
HY5 (IABW01205087)
PIF3 (IABW01141096)
GLK (IABW01095212)
PIF1 (IABW01005414)
GNC (IABW01148883)
Additional file 7: Figure S5

Supplement: Supplementary file 7 — Expression of Chl-related transcription factor genes in FW, FG, and FGD. Microarray data were obtained as described in Fig. 4. The GenBank accession number of each gene is indicated in parentheses. (PPTX 49 kb) [file 12870_2017_1156_MOESM7_ESM.pptx]

## Slide 1
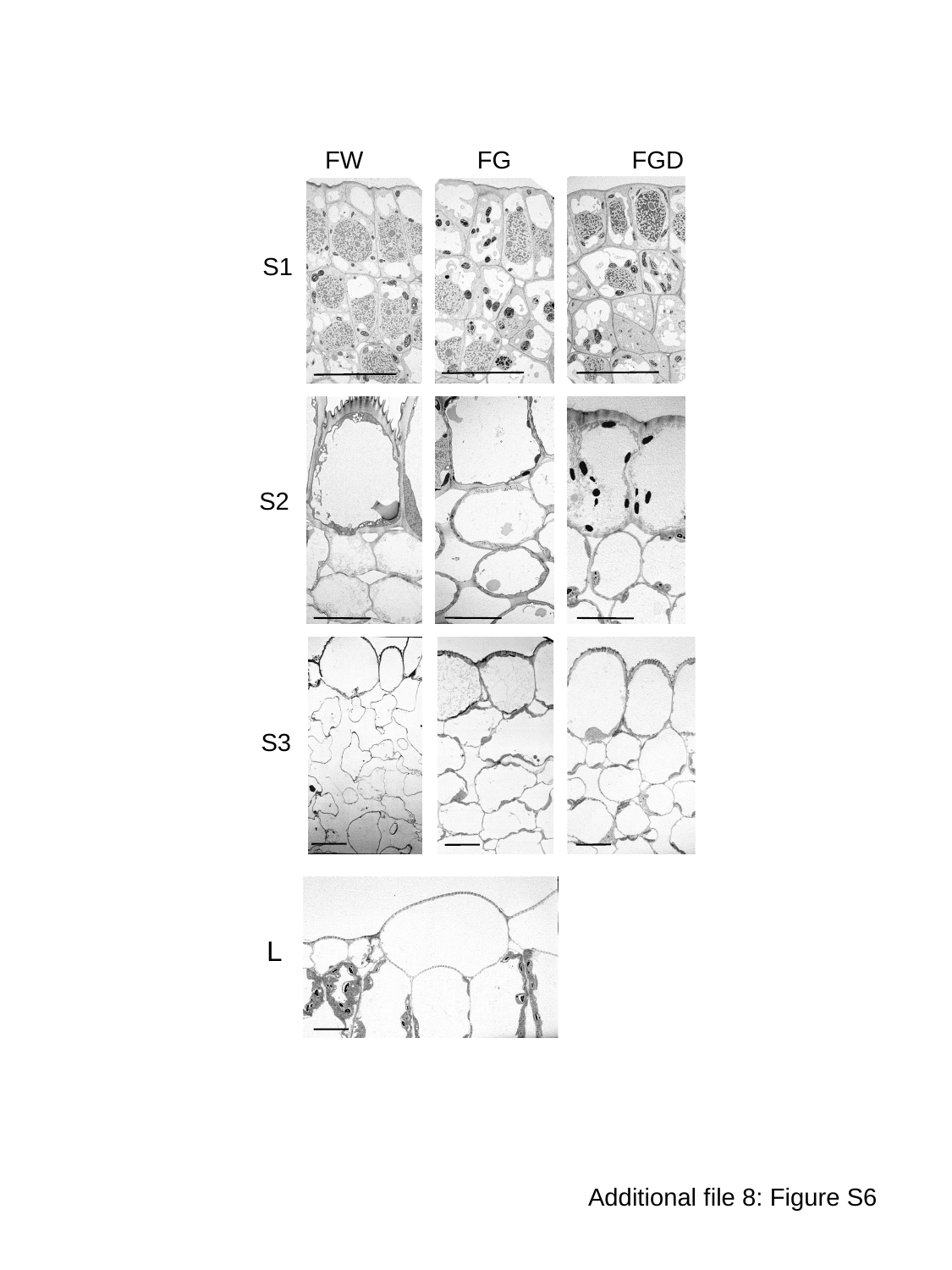

FW 　　 FG 　　 FGD
S1
S2
S3
L
Additional file 8: Figure S6

Supplement: Supplementary file 8 — Transmission electron microscopy at low magnification. Transverse sections of petals at stages 1 to 3 (S1 to S3), and leaves (L). The uppermost cell layer in each photograph is the epidermal cell layer. Bar = 20 μm. (PPTX 10712 kb) [file 12870_2017_1156_MOESM8_ESM.pptx]
