# Supplementary material for: Transcriptome analysis in petals and leaves of chrysanthemums with different chlorophyll levels
Source: BMC Plant Biol. 2017 Nov 15;17:202. doi: 10.1186/s12870-017-1156-6 (PMC5688696; doi:10.1186/s12870-017-1156-6)
Supplement: Supplementary file 3 — List of chlorophyll metabolic genes. (PPTX 69 kb) [file 12870_2017_1156_MOESM3_ESM.pptx]

## Slide 1
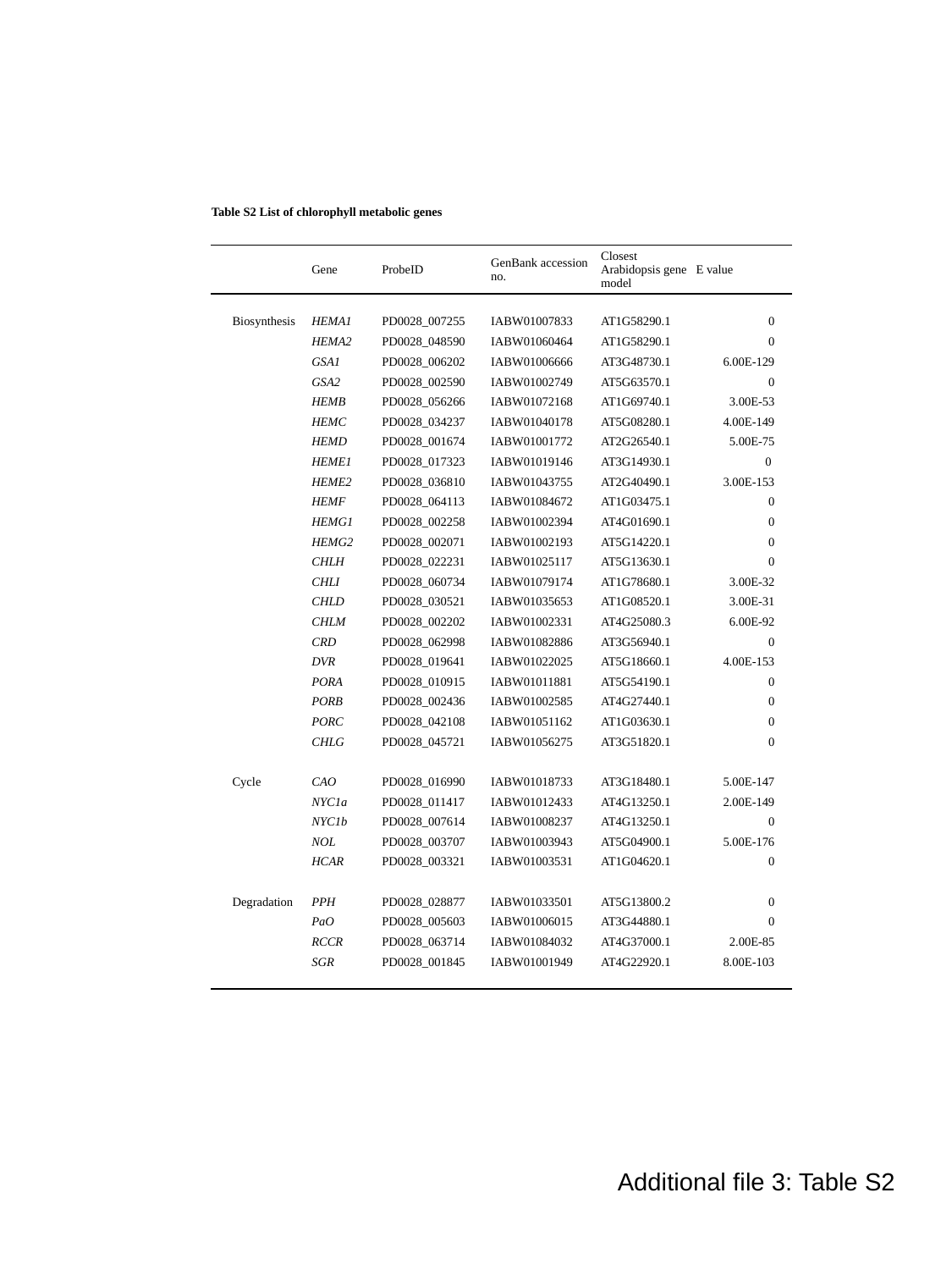

| Table S2 List of chlorophyll metabolic genes | | | | | | | |
| --- | --- | --- | --- | --- | --- | --- | --- |
| | | | | | | | |
| | | Gene | ProbeID | GenBank accession no. | Closest Arabidopsis gene model | E value | |
| | | | | | | | |
| | Biosynthesis | HEMA1 | PD0028\_007255 | IABW01007833 | AT1G58290.1 | 0 | |
| | | HEMA2 | PD0028\_048590 | IABW01060464 | AT1G58290.1 | 0 | |
| | | GSA1 | PD0028\_006202 | IABW01006666 | AT3G48730.1 | 6.00E-129 | |
| | | GSA2 | PD0028\_002590 | IABW01002749 | AT5G63570.1 | 0 | |
| | | HEMB | PD0028\_056266 | IABW01072168 | AT1G69740.1 | 3.00E-53 | |
| | | HEMC | PD0028\_034237 | IABW01040178 | AT5G08280.1 | 4.00E-149 | |
| | | HEMD | PD0028\_001674 | IABW01001772 | AT2G26540.1 | 5.00E-75 | |
| | | HEME1 | PD0028\_017323 | IABW01019146 | AT3G14930.1 | 0 | |
| | | HEME2 | PD0028\_036810 | IABW01043755 | AT2G40490.1 | 3.00E-153 | |
| | | HEMF | PD0028\_064113 | IABW01084672 | AT1G03475.1 | 0 | |
| | | HEMG1 | PD0028\_002258 | IABW01002394 | AT4G01690.1 | 0 | |
| | | HEMG2 | PD0028\_002071 | IABW01002193 | AT5G14220.1 | 0 | |
| | | CHLH | PD0028\_022231 | IABW01025117 | AT5G13630.1 | 0 | |
| | | CHLI | PD0028\_060734 | IABW01079174 | AT1G78680.1 | 3.00E-32 | |
| | | CHLD | PD0028\_030521 | IABW01035653 | AT1G08520.1 | 3.00E-31 | |
| | | CHLM | PD0028\_002202 | IABW01002331 | AT4G25080.3 | 6.00E-92 | |
| | | CRD | PD0028\_062998 | IABW01082886 | AT3G56940.1 | 0 | |
| | | DVR | PD0028\_019641 | IABW01022025 | AT5G18660.1 | 4.00E-153 | |
| | | PORA | PD0028\_010915 | IABW01011881 | AT5G54190.1 | 0 | |
| | | PORB | PD0028\_002436 | IABW01002585 | AT4G27440.1 | 0 | |
| | | PORC | PD0028\_042108 | IABW01051162 | AT1G03630.1 | 0 | |
| | | CHLG | PD0028\_045721 | IABW01056275 | AT3G51820.1 | 0 | |
| | | | | | | | |
| | Cycle | CAO | PD0028\_016990 | IABW01018733 | AT3G18480.1 | 5.00E-147 | |
| | | NYC1a | PD0028\_011417 | IABW01012433 | AT4G13250.1 | 2.00E-149 | |
| | | NYC1b | PD0028\_007614 | IABW01008237 | AT4G13250.1 | 0 | |
| | | NOL | PD0028\_003707 | IABW01003943 | AT5G04900.1 | 5.00E-176 | |
| | | HCAR | PD0028\_003321 | IABW01003531 | AT1G04620.1 | 0 | |
| | | | | | | | |
| | Degradation | PPH | PD0028\_028877 | IABW01033501 | AT5G13800.2 | 0 | |
| | | PaO | PD0028\_005603 | IABW01006015 | AT3G44880.1 | 0 | |
| | | RCCR | PD0028\_063714 | IABW01084032 | AT4G37000.1 | 2.00E-85 | |
| | | SGR | PD0028\_001845 | IABW01001949 | AT4G22920.1 | 8.00E-103 | |
| | | | | | | | |
| | | | | | | | |
Additional file 3: Table S2
