# Supplementary material for: Transcriptome analysis in petals and leaves of chrysanthemums with different chlorophyll levels
Source: BMC Plant Biol. 2017 Nov 15;17:202. doi: 10.1186/s12870-017-1156-6 (PMC5688696; doi:10.1186/s12870-017-1156-6)
Supplement: Supplementary file 5 — Expression of MYB113-like and anthocyanin biosynthesis genes in FW, FG, and FGD. Microarray data were obtained as described in Fig. 4. ANS, anthocyanidin synthase; CHI, chalcone isomerase; CHS, chalcone synthase; DFR, dihydroflavonol 4-reductase. GenBank accession number of each gene is indicated in parentheses. (PPTX 50 kb) [file 12870_2017_1156_MOESM5_ESM.pptx]

## Slide 1
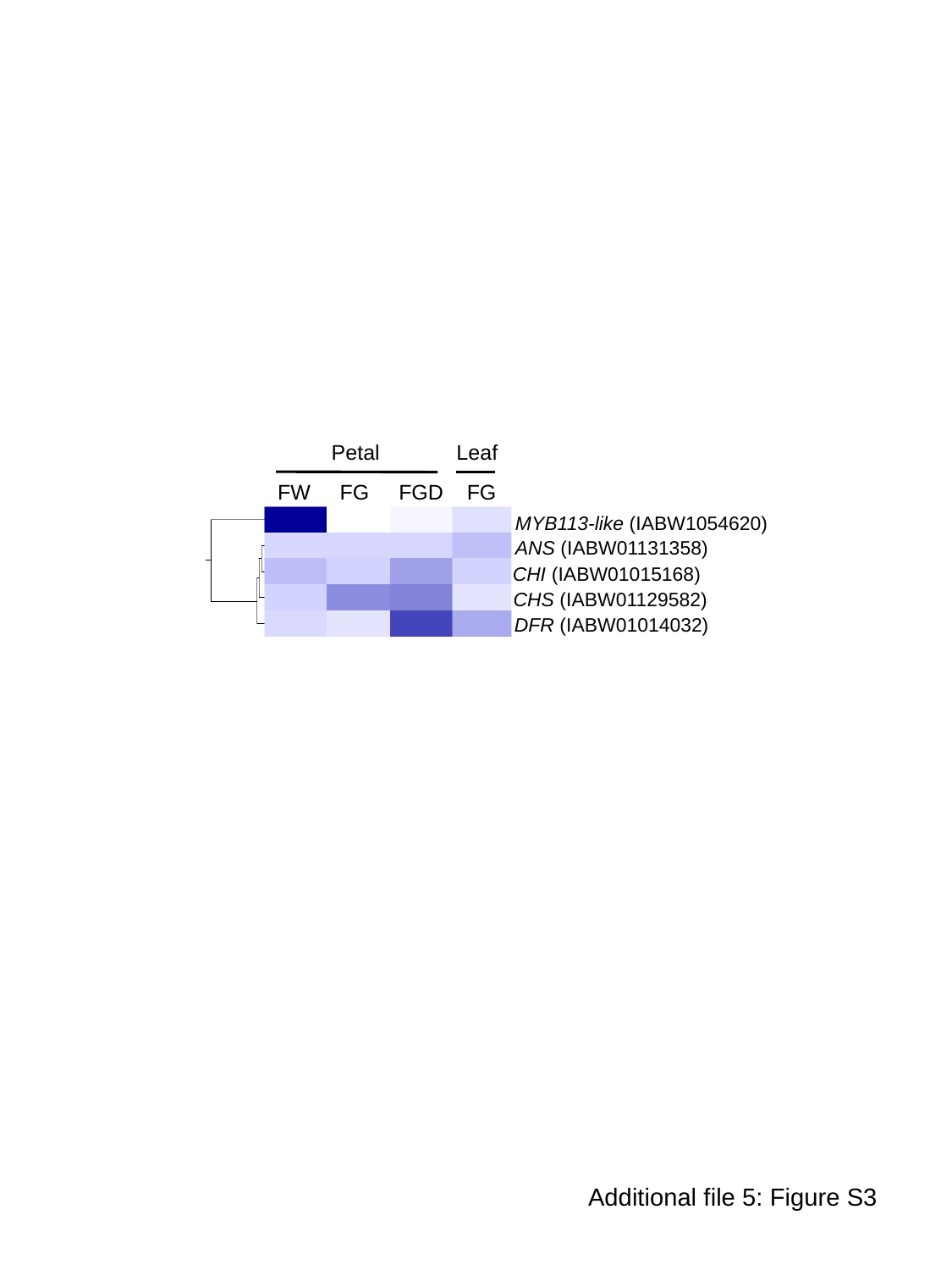

Petal Leaf
FW FG FGD FG
MYB113-like (IABW1054620)
ANS (IABW01131358)
CHI (IABW01015168)
CHS (IABW01129582)
DFR (IABW01014032)
Additional file 5: Figure S3
