# Supplementary material for: Transcriptome analysis in petals and leaves of chrysanthemums with different chlorophyll levels
Source: BMC Plant Biol. 2017 Nov 15;17:202. doi: 10.1186/s12870-017-1156-6 (PMC5688696; doi:10.1186/s12870-017-1156-6)
Supplement: Supplementary file 6 — RT-qPCR analysis of selected transcription factor genes in petals of white- and green-flowered chrysanthemum cultivars. Cultivar numbers are as in Additional file 4 Figure S2. A total of 11 genes were selected by microarray analysis (Tables 1 and 2). Three of them were differentially expressed between white and green petals (Fig. 7). The expression levels of the remaining 8 genes (presented in this Figure S4.), were not significantly different between white and green petals. Different letters indicate significant differences in Tukey–Kramer multiple-comparison test (P < 0.05). (PPTX 108 kb) [file 12870_2017_1156_MOESM6_ESM.pptx]

## Slide 1
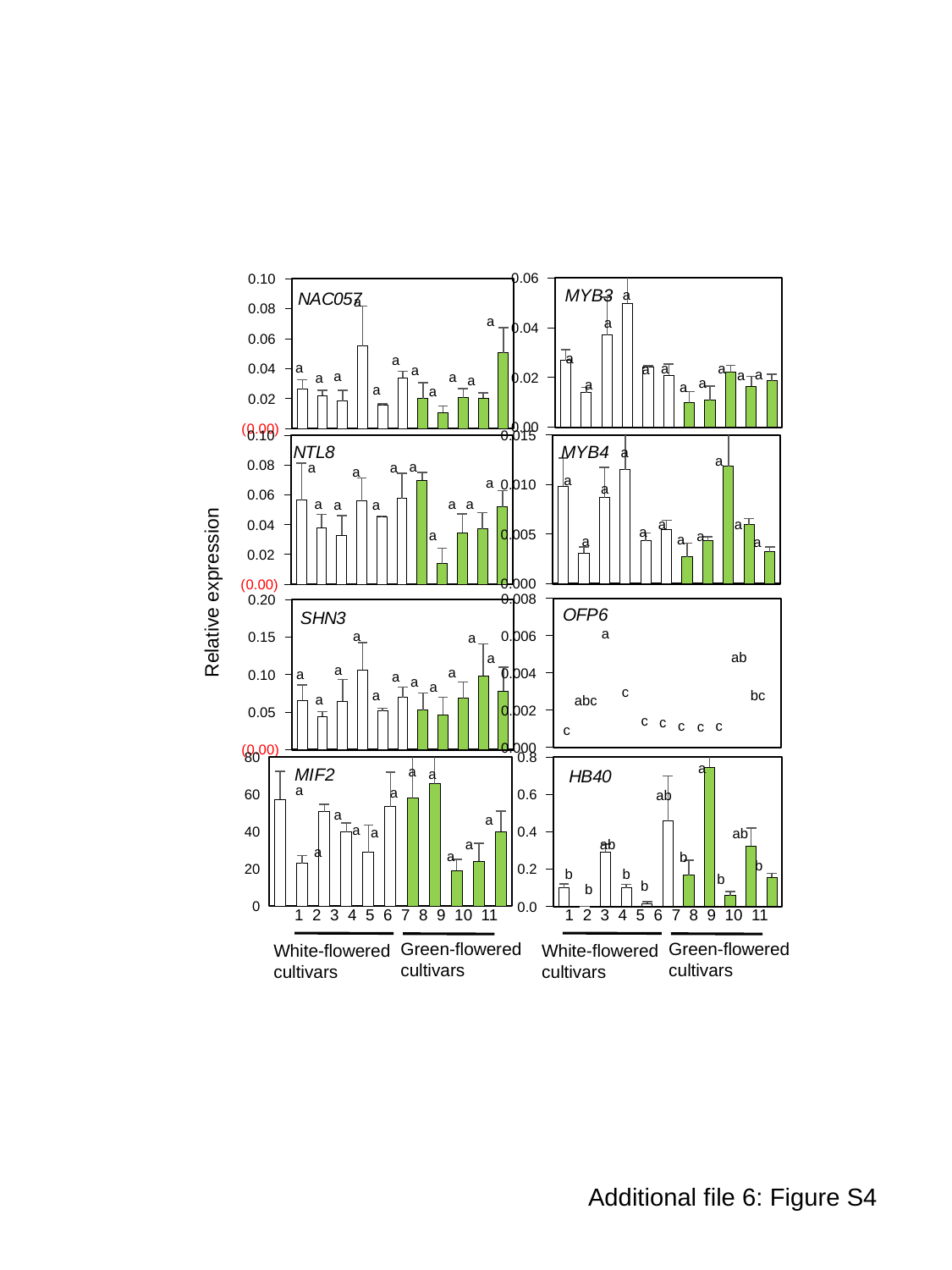

### Chart
| Category | |
|---|---|
| ES | 0.0267979429907711 |
| BT | 0.013892550963732 |
| SP | 0.0370576970001931 |
| SE | 0.049803196017203 |
| RD | 0.0240051525302874 |
| FR | 0.0208272045149333 |
| GN | 0.00978484679731614 |
| GR | 0.0108366380031902 |
| AG | 0.0220601315096935 |
| OL | 0.0164950460633182 |
| GL | 0.0187105854970616 |
### Chart
| Category | |
|---|---|
| ES | 0.026175368622257344 |
| BT | 0.02191784225255991 |
| SP | 0.018236588632932774 |
| SE | 0.05497648251205736 |
| RD | 0.015408897097465077 |
| FR | 0.03391620385384472 |
| GN | 0.020177490628597586 |
| GR | 0.010628334928706516 |
| AG | 0.020638506592969837 |
| OL | 0.02041869785736462 |
| GL | 0.05072489120058065 |a
a
a
a
a
a
a
a
a
a
a
a
a
a
a
a
a
a
a
a
a
a
### Chart
| Category | |
|---|---|
| ES | 0.00984053777715447 |
| BT | 0.00309807253194973 |
| SP | 0.00866872887085216 |
| SE | 0.0114908624458378 |
| RD | 0.00434344749491897 |
| FR | 0.00547878656090265 |
| GN | 0.00270096474896716 |
| GR | 0.00434678266823957 |
| AG | 0.0118622263750677 |
| OL | 0.00595172492193231 |
| GL | 0.00320246814172999 |
### Chart
| Category | |
|---|---|
| ES | 0.05653535901075185 |
| BT | 0.03800650542993547 |
| SP | 0.032850249227587835 |
| SE | 0.05594917377751847 |
| RD | 0.04502630322367889 |
| FR | 0.0576906080076284 |
| GN | 0.06974072542769444 |
| GR | 0.013853545031480974 |
| AG | 0.03448271606178744 |
| OL | 0.036994770038051884 |
| GL | 0.05191012860495527 |a
a
a
a
a
a
a
a
a
a
a
a
a
a
a
a
a
a
a
a
a
a
Relative expression
### Chart
| Category | |
|---|---|
| ES | 8.0186678363253e-05 |
| BT | 0.00179014562919148 |
| SP | 0.0053011393647256 |
| SE | 0.000251271049463481 |
| RD | 0.000498627231067348 |
| FR | 0.000472615805227801 |
| GN | 5.02151158396669e-05 |
| GR | 0.000150286840047549 |
| AG | 0.000186154134750561 |
| OL | 0.00436600712561556 |
| GL | 0.00110924098604039 |
### Chart
| Category | |
|---|---|
| ES | 0.06524946132439874 |
| BT | 0.04421080306422437 |
| SP | 0.06462303943492591 |
| SE | 0.10604254906867329 |
| RD | 0.05126228937145675 |
| FR | 0.069655656456139 |
| GN | 0.052356070685658174 |
| GR | 0.046285048926895396 |
| AG | 0.06831918361064789 |
| OL | 0.0981567891276809 |
| GL | 0.07779178508039776 |a
a
a
ab
a
a
a
a
a
a
a
c
bc
a
a
abc
c
c
c
c
c
c
### Chart
| Category | |
|---|---|
| ES | 0.101131636844714 |
| BT | 0.00149145644875029 |
| SP | 0.292547731127354 |
| SE | 0.0992306201068138 |
| RD | 0.017186412435601 |
| FR | 0.458728323850686 |
| GN | 0.167077365226152 |
| GR | 0.745651375661736 |
| AG | 0.0604440687435008 |
| OL | 0.324460567162002 |
| GL | 0.156806464961687 |
### Chart
| Category | |
|---|---|
| ES | 57.0730551464404 |
| BT | 22.781367965520257 |
| SP | 50.67391507466805 |
| SE | 39.578571439936105 |
| RD | 28.74979460134175 |
| FR | 53.40279982188991 |
| GN | 57.828521568079616 |
| GR | 65.579896412774 |
| AG | 18.94343346909285 |
| OL | 24.03382596674111 |
| GL | 39.80569282295456 |a
a
a
a
a
ab
a
a
a
a
ab
a
ab
a
a
b
b
b
b
b
b
b
1 2 3 4 5 6 7 8 9 10 11
1 2 3 4 5 6 7 8 9 10 11
Green-flowered
cultivars
White-flowered
cultivars
Green-flowered
cultivars
White-flowered
cultivars
Additional file 6: Figure S4
